# Six addiction components of problematic social media use in relation to depression, anxiety, and stress symptoms: a latent profile analysis and network analysis

**DOI:** 10.1186/s12888-023-04837-2

**Published:** 2023-05-08

**Authors:** Pu Peng, Yanhui Liao

**Affiliations:** 1 Department of Psychiatry, Sir Run Run Shaw Hospital, Zhejiang University School of Medicine, 3 East Qingchun Road, Hangzhou, 310016 Zhejiang China; 2 Department of Psychiatry, National Clinical Research Center for Mental Disorders, and National Center for Mental Disorders, The Second Xiangya Hospital of Central South University, Changsha, Hunan China

**Keywords:** Network analysis, Latent profile analysis, Problematic social media use, Depression, Anxiety, Stress, Components of addiction

## Abstract

**Backgrounds:**

Components of addiction (salience, tolerance, mood modification, relapse, withdrawal, and conflict) is the most cited theoretical framework for problematic social media use (PSMU). However, studies criticized its ability to distinguish problematic users from engaged users. We aimed to assess the association of the six criteria with depression, anxiety, and stress at a symptom level.

**Methods:**

Ten thousand six hundred sixty-eight participants were recruited. Bergen Social Media Addiction Scale (BSMAS) was used to detect six addiction components in PSMU. We applied the depression-anxiety-stress scale to assess mental distress. Latent profile analysis (LPA) was conducted based on BSMAS items. Network analysis (NA) was performed to determine the symptom-symptom interaction of PSMU and mental distress.

**Results:**

(1) Social media users were divided into five subgroups including occasional users (10.6%, *n* = 1127), regular users (31.0%, *n* = 3309), high engagement low risk users (10.4%, *n* = 1115), at-risk users (38.1%, *n* = 4070), and problematic users (9.8%, *n* = 1047); (2) PSMU and mental distress varied markedly across subgroups. Problematic users had the most severe PSMU, depression, anxiety, and stress symptoms. High engagement users scored high on tolerance and salience criteria of PSMU but displayed little mental distress; (3) NA showed conflict and mood modification was the bridge symptoms across the network, while salience and tolerance exhibited weak association with mental distress.

**Conclusions:**

Salience and tolerance might not distinguish engaged users from problematic users. New frameworks and assessment tools focusing on the negative consequences of social media usage are needed.

**Supplementary Information:**

The online version contains supplementary material available at 10.1186/s12888-023-04837-2.

## Background

With the rapid development of social media, problematic social media use (PSMU) has attracted increasing attention [1–3]. According to a recent meta-analysis, the global prevalence of PSMU in social media users is 17.42% [4]. While there is no universally accepted definition of PSMU, most researchers recognize it as excessive use of social media, which leads to negative consequences on the user’s functioning and psychological well-being [1].

### Six addiction components of problematic social media use

The theoretical frameworks for PSMU remain undetermined. To date, the most cited theory for PSMU is the six components of addiction proposed by Griffiths, which also serves as the theoretical basis for most of the PSMU assessment tools [2, 5]. It conceptualizes PSMU as a combination of salience (spending a lot of time thinking about or planning to use social media), tolerance (need to spend an increased amount of time on social media), mood modification (using social media to forget about the emotional problem), relapse (failure in cutting down the social media usage), withdrawal (feeling troubled when unable to use social media), and conflict (negative impacts of excessive usage of social media on the user’s life) [6]. However, emerging studies suggest that the components of the addiction model might not be suitable for PSMU [1]. They criticize the ability of salience and tolerance criteria to distinguish problematic users from high engagement users, which might pathologize normal social media use [1]. For example, a recent study by Fournier et al. found that the six addiction components of PSMU failed to form a unitary construct [7]. They reported that salience and tolerance criteria showed no relationship with psychopathological symptoms, suggesting that these two criteria might be peripheral features of PSMU. Our previous study also demonstrates that salience and tolerance symptoms contribute little to PSMU according to the machine learning procedure [8].

Numerous studies report that PSMU exhibits substantial association with mental distress such as depression, anxiety, and impaired sleep [9–14]. However, the assessment of the relationship between PSMU and mental distress in previous studies has been predominantly limited to the use of total scores of measurement instruments (e.g., calculating total scores on six addiction components and interpreting the total score as the severity of PSMU). The potential heterogeneity of the symptoms might be obscured [15]. Describing the unique association between the six addiction components in PSMU and mental distress might help better understand their relationship and conceptualize PSMU.

### Latent profile analysis and network analysis

Latent profile analysis (LPA) is a widely used, person-centered, categorical latent variable modeling approach [16]. LPA could identify latent subpopulations who shared similarities in a certain set of variables. In the case of PSMU, LPA allows for uncovering how social media users can be similarly “grouped” or “profiled” based on their PSMU symptoms and determining the similarities and differences across the profiles [17]. Network analysis (NA) is an emerging analysis tool for clarifying whether and how symptoms interact and reinforce one another [18]. In particular, NA offers a new angle on comorbidity by identifying the “bridge symptoms” across distinct psychiatric disorders [19, 20]. Both LPA and NA have been widely used in evaluating the association of mental distress with internet gaming disorder (IGD) or internet addiction [21–24]. However, studies on the PSMU via the two tools were very limited.

### The present study

Hence, our study aimed to: (1) determine the subtypes of Chinese social media users based on their PSMU symptoms (i.e., six components of addiction in PSMU); (2) to evaluate the mental distress (depression, anxiety, and stress) across different subtypes; and (3) to assess the bridge symptoms which linked PSMU and mental distress.

## Method

### Participants and procedure

The present study is a part of a large project on mental health and changes in the usage of internet gaming [25], social media [26], alcohol [27], and tobacco [28] in the Chinese general population in the late stage of the COVID-19 pandemic. The online survey was conducted from May 2020 to August 2020. Participants across China were recruited through snowball sampling via social media such as WeChat. Participants completed the questionnaire on WenJuanXin (www.wjx.cn, the most popular online survey platform in China), and could only submit the questionnaire after responding to all questions. All participants aged over 18 were eligible for this study. Before the start of the survey, participants gave their informed consent. Ethical approval was obtained from the Ethics Committee of Sir Run Run Shaw hospital (NO. 20200505–33).

### Measurements

Basic information including age, gender, education, occupation, partnership status, and residency were collected through self-designed questionnaire.

The changes in social media were assessed through self-reported questionnaires [26]. Participants were asked to recall their average time on social media (including but not limited to WeChat, QQ, Weibo, and TikTok) on weekdays and weekends both before and after the COVID-19 pandemic. Similar questionnaires were adapted in studies regarding the changes in internet use during the pandemic [29]. Weekly time on social media was calculated as (daily using time spent on a working day × 5) + (daily using time spent on a weekend day × 2).

We used the Bergen Social Media Addiction Scale (BSMAS) to assess PSMU, which has gained strong validity in the Chinese population [30, 31]. BSMAS is designed based on the components of addiction theory. It assesses the six addiction criteria including salience, tolerance, mood modification, relapse), withdrawal, and conflict of PSMU. BSMAS applied a 5-Likert scale and participants rated their frequency of symptoms ranging from 1 (very rarely) to 5 (very often). Higher scores indicated more severe PSMU. The Cronbach ‘s α of BSAMS was 0.862 in our study, suggesting its high internal consistency.

Depression, anxiety, and stress were assessed via the 21-item Depression-Anxiety-Stress Scale (DASS-21). It has three subscales and included 21 items. A 4-Likert scale ranging from 0 (Not at all) to 3 (Almost always) was used. The DASS-21 was widely used in the Chinese population and had gained strong validity [32]. The total scores of each subscale were calculated by multiplying the raw total scores by 2. The cutoff point of 14, 10, and 19 was used to screen for clinically relevant depression, anxiety, and stress symptoms. The Cronbach ‘s α of DASS-21, DASS-21 depression subscale, DASS-21 anxiety subscale, DASS-21 stress subscale was 0.956, 0.897, 0.876, and 0.887, respectively.

### Statistical analysis

We presented the continuous data as the median and interquartile range (IRQ; 25–75%) and the categorical data as frequency and percentage. All the tests were 2-tailed with *p* < 0.05 implying statistically significant. All the statistical analysis was done in R (ver.4.2.0).

### Latent profile analysis

We conducted the latent profile analysis (LPA) based on the scores of the items of the six components of BSMAS via the R package “tidyLPA”. We estimated the model with 2–5 latent profiles. The following metrics of each model were calculated: the Bayesian information criterion (BIC), Akaike’s information criterion (AIC), and entropy [8]. The model was selected based on previous guidelines [16]. Specifically, the following indicators were considered: (1) lower relative fit information criteria, which includes lower AIC and BIC, (2) high entropy of at least 0.8, and (3) the results of bootstrap likelihood ratio (BLRT). BLRT *p*-value less than 0.05 indicated a significant improvement in model fit when compared to the solution with one less class. We compared the inter-group differences in demographic information, social medial variables, and mental health through the Chi-square test and Wilcoxon rank-sum test. We further conducted a multivariate logistic regression to determine the correlates of each subgroup of social media users.

### Network analysis

We estimated the network of PSMU, depression, anxiety, and stress symptoms via the EBICglasso model, which was widely used in psychological networks [33]. The R package “bootnet” and “qgraph” was used to estimate and visualize the network. In the network, each symptom was represented as a “node” and the unique association within the symptoms after controlling for other nodes in the network was represented as an “edge”. Thicker edges suggested a stronger association. Red and blue edges suggested positive and negative associations, respectively. We calculated the expected influence (EI) and bridge expected influence (BEI) to determine the central and bridge symptoms within the PSMU-Depression-Anxiety -Stress network [19]. Nodes with higher BEI were recognized as bridge symptoms that linked the PSMU community and the depression-anxiety-stress community. We used a case-dropping bootstrap approach to test the stability of BEI and EI [33]. The correlation stability coefficient (CS-C) represented the stability of the network. A CS-C higher than 0.5 was considered to be good. Nonparametric bootstrapping with 1000 bootstrap samples was performed to test the accuracy of the edges within the network.

## Result

### Sample Characteristics

A total of 11, 029 participants were recruited. After removing responses with a logic error such as abnormal age or daily social media use and responses which were submitted within 2 min or longer than one hour, 10, 668 validated responses were included in the final analysis (Table 1). The majority of the participants were female (6099, 57%), employed (8307, 78%), married (6378, 60%), had a bachelor’s degree (7167, 67%), and lived in the urban (8027, 75%). The median weekly social usage was 960 (420, 1680) minutes, and 3070 (29%) of the participants increased their social media usage by 3.5 h per week during the pandemic. The prevalence of clinically relevant depression, anxiety, and stress was 18%, 26%, and 10%, respectively.

**Table 1:** Sample characteristics

**Variable**	Overall, *N* = 10,668^1^	Occasional user, *N* = 1,127^1^	Regular user, *N* = 3,309^1^	High engagement low risk users, *N* = 1,115^1^	At-risk user, *N* = 4,070^1^	Problematic user, *N* = 1,047^1^	*p*-value^2^
**Gender**							< 0.001
Male	4,569 (43%)	502 (45%)^a,b,c^	1,391 (42%)^b,c^	508 (46%)^a,b^	1,655 (41%)^c^	513 (49%)^a^	
Female	6,099 (57%)	625 (55%)	1,918 (58%)	607 (54%)	2,415 (59%)	534 (51%)	
**Age, year**	33 (25, 40)	37 (28, 43)^a^	33 (25, 40)^b^	34 (27, 41)^c^	31 (24, 39)^d^	32 (25, 39)^d^	< 0.001
**Education**							0.002
Below college	858 (8.0%)	123 (11%)^b^	285 (8.6%)^a,b^	77 (6.9%)^a^	291 (7.1%)^a^	82 (7.8%)^a,b^	
College	7,167 (67%)	737 (65%)	2,211 (67%)	739 (66%)	2,787 (68%)	693 (66%)	
Master or above	2,643 (25%)	267 (24%)	813 (25%)	299 (27%)	992 (24%)	272 (26%)	
**Employed**	8,307 (78%)	964 (86%)^a^	2,601 (79%)^c^	942 (84%)^a^	2,997 (74%)^b^	803 (77%)^b,c^	< 0.001
**Married status**							< 0.001
Married	6,378 (60%)	774 (69%)^b^	2,033 (61%)^a,d^	724 (65%)^a,b^	2,237 (55%)^c^	610 (58%)^c,d^	
Single	3,997 (37%)	312 (28%)	1,186 (36%)	358 (32%)	1,741 (43%)	400 (38%)	
Divorced	293 (2.7%)	41 (3.6%)	90 (2.7%)	33 (3.0%)	92 (2.3%)	37 (3.5%)	
**Living in city**	8,027 (75%)	861 (76%)^a,b^	2,434 (74%)^b^	879 (79%)^a^	3,060 (75%)^a,b^	793 (76%)^a,b^	0.008
**Social media usage before the pandemic, min**	840 (420, 1,380)	420 (210, 840)^c^	690 (410, 1,110)^d^	900 (480, 1,500)^a^	840 (420, 1,440)^b^	960 (460, 1,850)^e^	< 0.001
**Social media usage after the pandemic, min**	960 (420, 1,680)	540 (210, 1,096)^b^	840 (420, 1,400)^c^	1,110 (630, 2,000)^a^	1,120 (560, 1,980)^a^	1,400 (700, 2,290)^d^	< 0.001
**Social time change**	3,070 (29%)	182 (16%)^c^	794 (24%)^d^	330 (30%)^a^	1,375 (34%)^a,b^	389 (37%)^b^	< 0.001
**BSAMS scores**	16 (12, 19)	7 (6, 9)^c^	13 (12, 14)^d^	15 (14, 16)^a^	18 (17, 20)^b^	24 (23, 25)^e^	< 0.001
**Stress scores**	4(1, 7)	1 (0, 3)^c^	3 (1, 6)^a^	3 (1, 6)^a^	5 (2, 7)^b^	8 (4, 12)^d^	< 0.001
**Stress**	1,117 (10%)	34 (3.0%)^c^	153 (4.6%)^a,c^	65 (5.8%)^a^	499 (12%)^b^	366 (35%)^d^	< 0.001
**Anxiety scores**	2 (0, 5)	0 (0, 2)^c^	1 (0, 3)^a^	1 (0, 3)^a^	3 (1, 6)^b^	6 (2, 9)^d^	< 0.001
**Anxiety**	2,774 (26%)	105 (9.3%)^c^	559 (17%)^a^	167 (15%)^a^	1,340 (33%)^b^	603 (58%)^d^	< 0.001
**Depression scores**	2 (0, 5)	0 (0, 2)^c^	2 (0, 4)^d^	1 (0, 3)^a^	3 (1, 6)^b^	6 (2, 10)^e^	< 0.001
**Depression**	1,911 (18%)	70 (6.2%)^a^	344 (10%)^b^	99 (8.9%)^a,b^	926 (23%)^c^	472 (45%)^d^	< 0.001

^1^n (%); Median (IQR)

^2^Pearson's Chi-squared test; Kruskal–Wallis rank sum test

Different subscript letters (a, b, c, d, e) in the same row reflect statistically significant (*p* < 0.05) difference

### Latent profile analysis

**Fig. 1:**
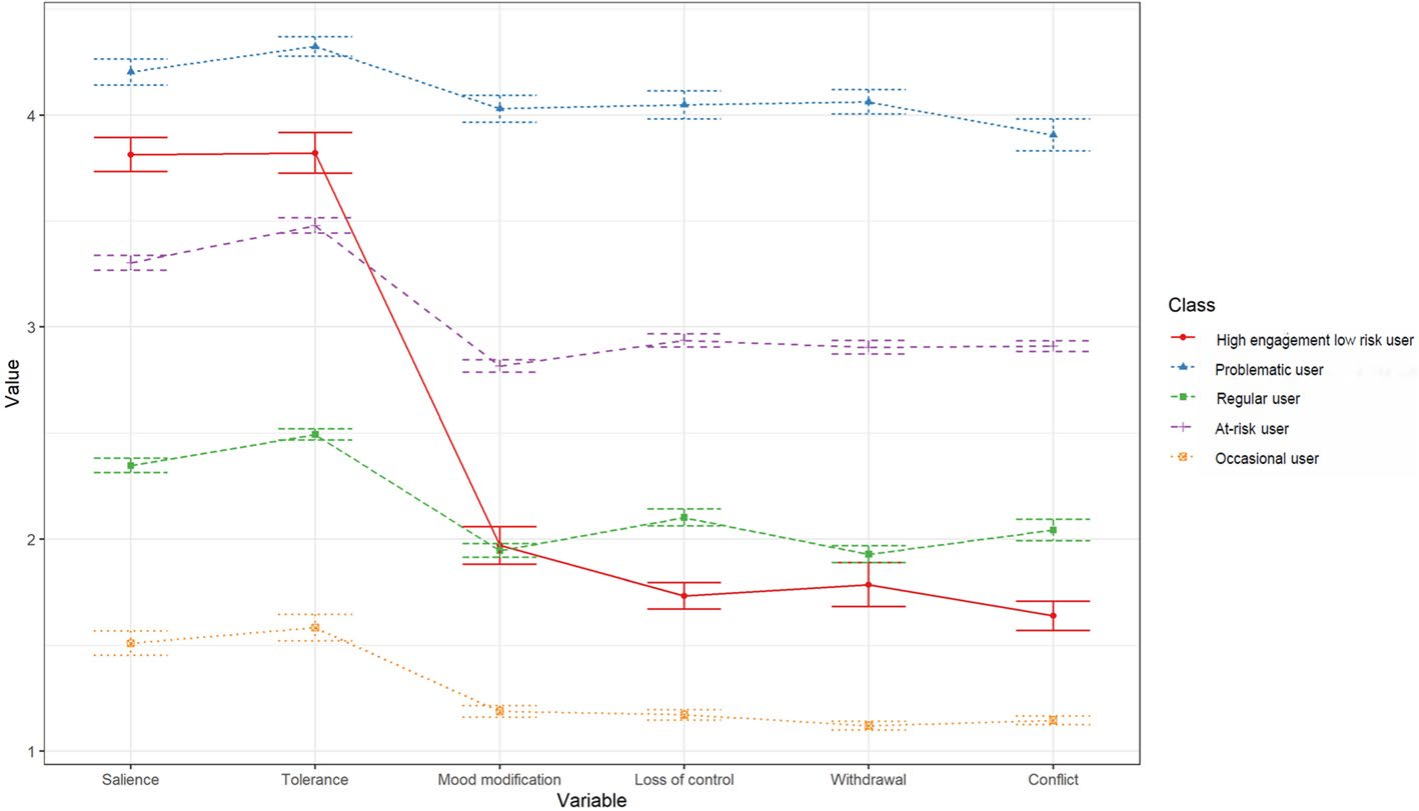
The five subgroups of social media users. X-axis represents the BSAMS items. Y-axis represents the scores of each BSAMS item

Table 2 describes the class solutions from 2 to 5, and a five-class model was considered the best fit. It has the lowest AIC and BIC and has good entropy. The LMR-LRT indicates that the five-class model was statistically greater than a four-class model. Figure 1 illustrated the five-class model of PSMU. The first class (“occasional user”) and the second class (“regular user”) contained 1127 (10.6%), and 3309 (31.0%) participants, respectively. They generally reported “very rarely” and “rarely” in all BSMAS items. Participants in class 3 were characterized by reporting “sometimes” in all BSMAS items, and thus class 3 (4070, 38.1%) was named the “at-risk user” group. Class 4, named the “high engagement low risk user” group, contained 1115 members who rated “often” in “salience” and “tolerance” symptoms but scored “rarely” in other items of BSMAS. 1047 participants were included in the fifth class, named “problematic user” groups. They scored highest on the BSMAS items as “often” or “very often”.

**Table 2:** Fit indices of the latent profile models

Classes	LogLike	AIC	BIC	Entropy	BLRT_p
2	-85984.5	172007	172145.2	0.810	< 0.01
3	-83030.4	166112.8	166301.9	0.828	< 0.01
4	-81811.2	163688.4	163928.4	0.810	< 0.01
**5**	**-80769.1**	**161618.2**	**161909.2**	**0.808**	** < 0.01**

*AIC* Akaike information criterion, *BIC* Bayesian information criterion, *BLRT* bootstrap likelihood ratio test, *p* < 0.05 suggesting significant better performance

There was a significant difference in demographic characteristics, social media usage, and mental health conditions among the five classes. Participants in the problematic user class spent significantly more time on social media both before and during the pandemic and were at a much higher risk for depression (45%), anxiety (58%), and stress (35%) compared with the other four classes. In contrast, regular users and occasional users tended to spend less time on social media and scored lower in depression, anxiety, and stress.

Participants in the “high engagement low risk user” and “at-risk user” groups had similar weekly social media time, which was higher than that of “occasional user” and “regular user”. However, “at-risk users” members were more prone to depression (23% vs 8.9%), anxiety (33% vs 15%), and stress (12% vs 5.8%) than “High engagement low risk” members (all *p* < 0.05). Also, despite the high social media usage, “High engagement low risk” members were not at a higher risk for mental distress than “regular users” members.

Table 3 described the independent correlate of each subgroup identified by LPA. Compared to occasional user, anxiety and social media usage were significantly associated with all other four subgroups. Notably, problematic user was additionally related to age, education level, married status, depression, and stress. Age, gender, depression, and education level was independently associated with at-risk user.

**Table 3:** Correlates of the subgroups identified by latent profile analysis

Class	Characteristic	OR^a^	95% CI^a^	*p*-value
Problematic user^b^	Age	0.98	0.96, 0.99	< 0.001
	Depression	2.36	1.65, 3.36	< 0.001
	Anxiety	5.54	4.09, 7.48	< 0.001
	Stress	3.14	2.05,4.82	< 0.001
	Daily social media usage > 2 h after the pandemic	2.92	2.22,3.88	< 0.001
	Daily social media usage > 2 h before the pandemic	1.54	1.17, 2.04	0.002
	Being married^c^	0.63	0.54, 0.89	0.002
	Master or above^d^	1.83	1.27, 2.63	0.001
	College^d^	1.45	1.04, 2.01	0.027
At-risk user^b^	Age	0.98	0.97, 0.99	< 0.001
	Being female	1.16	1.01,1.33	0.041
	Depression	1.77	1.28, 2.44	< 0.001
	Anxiety	3.34	2.61, 4.40	< 0.001
	Daily social media usage > 2 h after the pandemic	2.12	1.71,2.62	< 0.001
	Daily social media usage > 2 h before the pandemic	1.48	1.19, 1.84	< 0.001
	Master or above^d^	1.48	1.13, 1.94	0.005
	College^d^	1.38	1.09, 1.76	0.009
High engagement low risk user^b^	Anxiety	1.63	1.18, 2.24	0.003
	Daily social media usage > 2 h after the pandemic	2.14	1.64,2.79	< 0.001
	Daily social media usage > 2 h before the pandemic	1.79	1.37, 2.33	< 0.001
	Master or above^d^	1.60	1.14, 2.27	0.007
	College^d^	1.45	1.06, 1.98	0.021
Regular user^b^	Age	0.987	0.978, 0.997	0.008
	Anxiety	1.88	1.44, 2.46	< 0.001
	Daily social media usage > 2 h after the pandemic	1.51	1.22, 1.87	< 0.001

^a^*OR* Odds Ratio, *CI* Confidence Interval

^b^Reference: Occasional user

^c^Reference: Being single

^d^Reference: education level below college`

### Network analysis

**Fig. 2:**
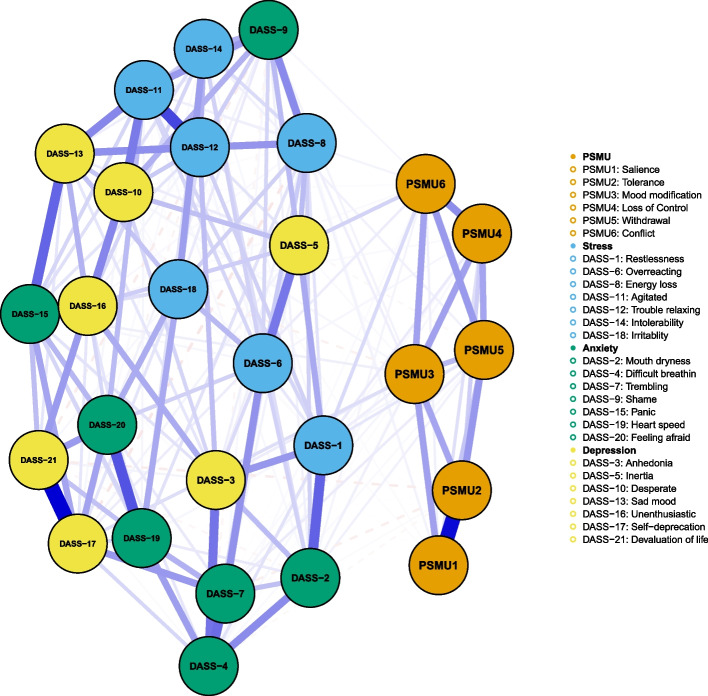
The network analysis of PSMU, depression, anxiety, and stress in at-risk users and problematic users. Orange, blue, green, and yellow nodes represent PSMU, stress, anxiety, and depression symptoms, respectively. Blue and red edges indicate positive and negative associations. Thicker edges suggest stronger associations

**Fig. 3:**
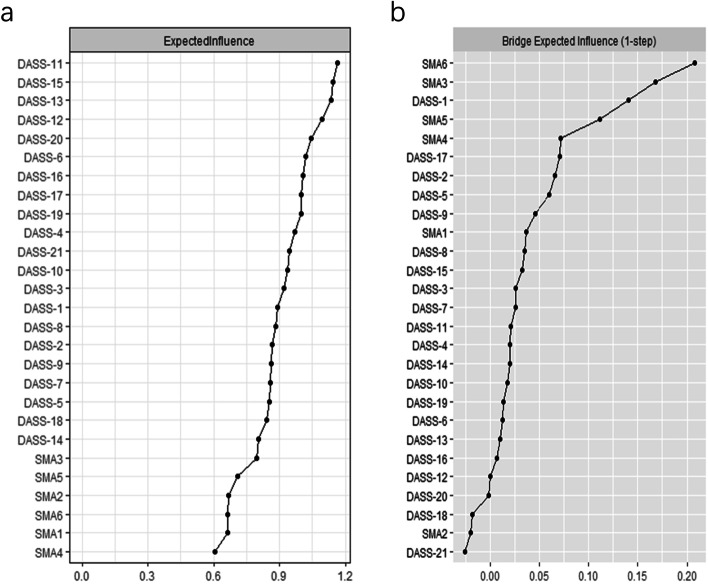
The centrality plot and bridge expected influence plot of the network. **a** The X-rays represented the expected influence of each node. Nodes with higher expected influence have stronger impact in other nodes within the network. **b** The X-rays represented the bridge expected influence of each node. Nodes with higher bridge expected influence are recognized as bridge symptoms that drove the comorbid depression and anxiety symptoms

We conducted the network analysis in the “problematic user” class and “at-risk user” class members, who were at a higher risk for PSMU, depression, anxiety, and stress. No items in BSMAS and DASS-21 were excluded for redundancy or low informativeness. Figure 2 illustrated the PSMU-Depression-Anxiety-Stress network, which had a density of 0.575 (202/351). The strongest edge within the network was BSMAS1 (Salience) and BSMAS2 (Tolerance), followed by DASS-17 (Self-deprecation) and DASS-21 (Devaluation of life). The centrality plot (Fig. 3a) suggested that DASS-11 (Agitated), DASS-15 (Panic), DASS-13 (Sad mood), and DASS-12 (Trouble relaxing) were the central symptoms of the PSMU-Depression-Anxiety-Stress network. BSMAS3 (Mood modification) exhibited the highest EI among PSMU symptoms. BSMAS6 (Conflict) was the bridge symptom that drove the comorbid mental symptoms and PSMU (Fig. 3b). It exhibited a substantial positive association with DASS-1 (Restlessness), DASS-5 (Inertia), and DASS-17 (Self-deprecation). Other bridge symptoms included BSMAS3 (Mood modification), DASS-1 (Restlessness), and BSMAS5 (Withdrawal). Of note, BSMAS2 (Tolerance) and BSMAS1 (Salience) displayed a very weak or negative association with depression, anxiety, and stress symptoms. The correlation matrix of BSMAS-DASS items were presented in Table S1.

The network had excellent stability and accuracy. Figure S1 displayed the central and bridge stability of the network. The CS-C of both nodes and bridge expected influence was 0.75, which suggested the two indexes remained correlated with the original data (*r* = 0.75) after dropping out 75% of the data. The 95% CI of the estimated edges was narrow, indicating excellent accuracy of the network (Figure S2).

## Discussion

In the present study, we assessed the relationship between six criteria of addiction in PSMU and mental distress via a person-centered and symptom-based approach among a large sample of Chinese social media users. The major findings included: (1) Based on their PSMU symptoms, social media users could be divided into five subgroups including occasional users, regular users, high engagement low risk users, at-risk users, and problematic users; (2) PSMU and mental distress symptoms varied markedly across the subgroups. Problematic users had the most severe PSMU, depression, anxiety, and stress symptoms. High engagement users scored high on tolerance and salience criteria of PSMU but displayed little mental distress; (3) network analysis showed conflict and mood modification was the bridge symptoms across the network, while salience and tolerance exhibited a rather weak association with mental distress. Taken together, our findings indicated that the tolerance and salience criteria of PSMU might not distinguish healthy users with high engagement from problematic users, which called for an updated framework of PSMU.

The 5-class model of social media users in the Chinese adult population perfectly replicated our previous findings in a large sample of Chinese college students [8], implying itself as a suitable classification for Chinese social media users. Almost half of the social media users were at high risk for PSMU, with 10% being problematic users and 38% being at-risk users. The prevalence of PSMU was highly variable in previous studies (range 3.5%-44.8%) [4, 8, 34, 35], which might result from the different study populations, measurement tools, and cutoff points. In line with previous studies [9–11, 34], problematic users spent more time on social media, scored higher in all PSMU symptoms, and had much higher levels of depression, anxiety, and stress. Hence, timely monitoring and intervention for PSMU are in urgent need.

Notably, we found a novel subgroup named high engagement low risk users, which was rarely reported in previous studies on internet addiction and IGD [24, 36–38]. They were characterized by a high level of salience and tolerance and scored low in other PSMU symptoms, which suggested that they were highly passionate social media users without suffering from the negative consequences of high social media usage. While their social media usage and BSMAS scores were higher than regular and occasional users, they didn’t exhibit worse depression, anxiety, and stress symptoms. This finding suggested the two criteria might be an indicator of high engagement in social media. Fournier et al. reported similar results with ours, finding that salience and tolerance were peripheral rather than central components of PSMU [7]. Studies in IGD also found that the two criteria had a low ability to distinguish normal and passionate users from problematic and addictive users [39–43]. Hence, a further theoretical framework for PSMU should focus more on the negative consequences of social media usage, which might help avoid pathologizing social media usage.

The NA provided new evidence about the relationship between PSMU and mental distress from a symptom perspective. In line with our LPA results, we found salience and tolerance exhibited a very weak association with mental distress, which was in accordance with that of Yue et al. in the association between internet addiction and depression symptoms [44]. Studies from other cultural backgrounds reported similar results. For example, a network analysis of PSMU and psychiatric symptoms in Italian social media users reported that salience and tolerance showed no relationship with psychiatric symptoms [7]. Cheng et al. also reported that these two symptoms displayed rather weak relationship with the depression in samples from the United Kingdom and the United States [34]. Our study suggested that conflict and mood modification were the bridge symptoms that drove the comorbidity of PSMU and mental distress. This finding supported two widely accepted conceptual frameworks in the association of problematic internet use and mental distress [30]. The first framework was the cognitive-behavioral model of pathological Internet use proposed by R.A. Davis, which suggested that mental distress was vital in the establishment of pathological internet use [45]. Users with mental distress tended to use the internet as a coping strategy and were more prone to problematic internet use. Another framework suggested that excessive internet use might contribute to mental distress due to disruption and impairment in real life [46]. While our study was cross-sectional and therefore could not identify the causal relationship between mental distress and PSMU, the NA indicated that both frameworks might be suitable for understanding their relationships. Restlessness (i.e., finding it hard to wind down) was another bridge symptom within the network, which exhibited a positive association with mood modification, relapse, conflict, and withdrawal symptoms. Further longitudinal studies are still warranted to confirm our findings.

While our studies provided novel insights into the relationship between PSMU and mental distress, there were several limitations. Firstly, the cross-sectional study design prohibited us from developing the causal relationship between PSMU and mental distress. Also, the data was collected during the COVID-19 pandemic. It is unclear whether and how the pandemic might affect our results. However, both the results of LPA and NA has successfully replicated previous studies prior to the pandemic [7, 8]. Hence, it is likely that the impact of the pandemic on our survey results might be modest. Further studies are needed to verify our findings. Secondly, the sample was collected via online may limit its representativeness of the Chinese population. However, this large sample size study recruited participants from 32 provinces in China, which enhances the generalizability of our findings; the demographic characteristics (such as age, sex ratio, education level, and employment status) [47, 48] and daily social media use [49] were similar to those of other large-scale representative studies in China. Moreover, the prevalence of PSMU in our sample was consistent with other epidemiological studies during the pandemic, indicating reasonable representativeness of our sample [50]. Another limitation was the application of self-reported questionnaire rather than a clinical interview. PSMU was assessed with one single scale, which might fail to cover several important dimensions of PSMU such as function impairment, displacement, and deception [5]. In addition, PSMU may reflect welfare problems through a proxy such as loneliness, which was not measured in our study [51, 52]. The association of salience and tolerance symptoms with these proxies remained unknown, which should be reminded in further studies.

## Conclusion

Almost half of the social media users were at high risk for PSMU, who were more prone to depression, anxiety, and stress. Both LPA and NA suggested that tolerance and salience criteria exhibited a weak association with mental distress and might not distinguish healthy and engaged users from problematic users. Further theoretical frameworks and assessment tools for PSMU, which focus more on the negative consequences of PSMU, are needed to avoid the pathologization of normal usage of social media.

## Supplementary Information

**Additional file 1: Supplementary Figure 1.** The stability of the network. **Supplementary Figure 2.** The accuracy of the edges estimated.

**Additional file 2: Supplementary Table 1.** Correlation matrix of BSMAS and DASS-21 items.

## Data Availability

The datasets used and/or analyzed during the current study are available from the corresponding author on reasonable request.

## Abbreviations

BSAMS: Bergen Social Media Addiction Scale
DASS-21: 21-Item Depression Anxiety Stress Scales
PSMU: Problematic social media usage
LPA: Latent profile analysis
NA: Network analysis
BIC: Bayesian information criterion
AIC: Akaike’s information criterion
BLRT: Bootstrap likelihood ratio
EI: Expected influence
CS-C: Correlation stability coefficient
BEI: Bridge expected influence
