# Supplementary material for: Six addiction components of problematic social media use in relation to depression, anxiety, and stress symptoms: a latent profile analysis and network analysis
Source: BMC Psychiatry. 2023 May 8;23:321. doi: 10.1186/s12888-023-04837-2 (PMC10166459; doi:10.1186/s12888-023-04837-2)
Supplement: Supplementary file 1 — Additional file 1: Supplementary Figure 1. The stability of the network. Supplementary Figure 2. The accuracy of the edges estimated. [file 12888_2023_4837_MOESM1_ESM.docx]

**Supplementary materials for “Six addiction components of problematic social media use in relation to depression, anxiety, and stress symptoms: a latent profile analysis and network analysis”**

**Supplementary Figure 1** The stability of the network

**Supplementary Figure 2** The accuracy of the edges estimated

**Supplementary Table 1** Correlation matrix of BSMAS and DASS-21 items

**Supplementary Figure 1** The stability of the network


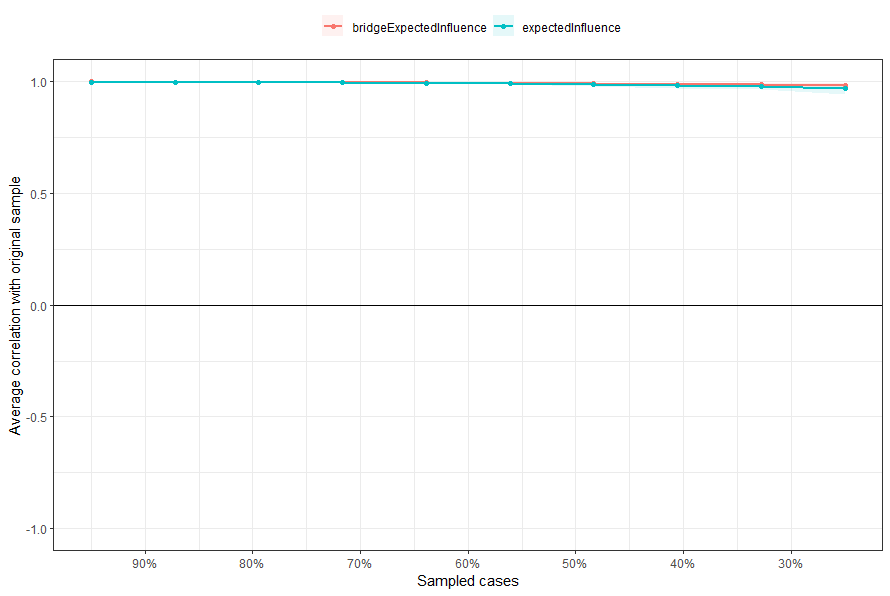


**Supplementary Figure 1** The stability of central and bridge expected influence by case-dropping bootstrap. The CS-C for the node and bridge expected influence was 0.672 and 0.75, respectively.

**Supplementary Figure 2** The accuracy of the edges estimated


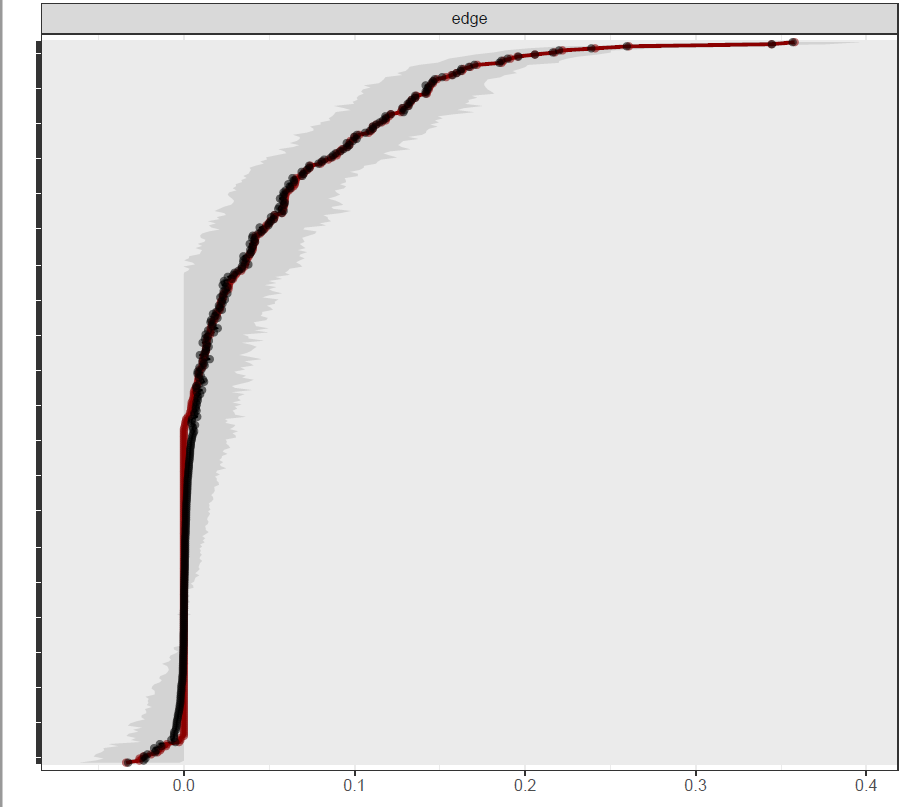


**Supplementary Figure 2** The accuracy of the edges estimated. The grey area represents the bootstrap 95% confidence interval.
